# Small worms of Sthenelanellinae, Pholoinae and Pisioninae (Annelida, Sigalionidae) from the Tropical Northwestern Atlantic

**DOI:** 10.7717/peerj.15005

**Published:** 2023-03-08

**Authors:** Christopher Cruz-Gómez

**Affiliations:** Departamento de Sistemática y Ecología Acuática, El Colegio de la Frontera Sur, Chetumal, Quintana Roo, Mexico

**Keywords:** Morphology, New species, Polychaeta, Sthenelanella, Taylorpholoe, Tropical Northwestern Atlantic

## Abstract

The family Sigalionidae is characterized, among other features, by including scale worms with large bodies. However, among sigalionids, the subfamilies Sthenelanellinae, Pholoinae, and Pisioninae stand out by their small representatives with few segments and fragile bodies. In the Tropical Northwestern Atlantic, which includes part of the Gulf of Mexico and the Caribbean Sea, these subfamilies have been rarely studied, with few species recorded, and questionable records. This contribution aims to improve the knowledge of sthenelanellins, pholoins, and pisionins in the region through a faunistic study based on material from two Mexican scientific collections: the Reference Collection of Laboratorio de Biodiversidad y Cambio Climático (BIOMARCCA) and the Reference Collection of Benthos (ECOSUR) of El Colegio de la Frontera Sur. *Pisione wolfi* is confirmed from the Tropical Northwestern Atlantic, and three new species are described: *Sthenelanella pechi* sp. nov., *S. sarae* sp. nov. and *Taylorpholoe anabelae* sp. nov. A key to all *Sthenelanella* and *Taylorpholoe* species is also included.

## Introduction

Sigalionids are recognized as relatively large scale worms, most belonging to the subfamilies Sigalioninae Kinberg (1856) or Pelogeniinae Chamberlin (1919). However, small specimens can also be found within the group, particularly in three subfamilies: Sthenelanellinae Gonzalez et al. (2018), Pholoinae Kinberg (1858) and Pisioninae Ehlers (1901). Until recently, the latter two were considered two distinct families, but studies based on morphological and molecular data led both families to be suppressed as two subfamilies belonging to Sigalionidae (Struck, Purschke & Halanych, 2005; Wiklund et al., 2005; Norlinder et al., 2012; Gonzalez et al., 2018; Zhang et al., 2018).

The members of these three subfamilies are typically recognized as small fragile worms with heterogeneous morphology. In the Tropical Northwestern Atlantic, the study of sthenelanellins, pholoins, and pisionins has been scarce with few species recorded.

The sthenelanellins are poorly known in the region, with only one species recorded, *Sthenelanella* sp. A by Wolf (1984) from the Gulf of Mexico. Regarding pholoins, three species have been recorded: *Taylorpholoe hirsuta* (Rullier & Amoureux, 1979) described from northeastern Brazil, and widely recorded in the Tropical Northwestern Atlantic (Pettibone, 1992; San Martín, Aguirre & Baratech, 1986). Also *Pholoe minuta* (Fabricius, 1780), described from Greenland, and recorded in Belize (Young & Young, 1982); and *Pholoides bermudensis* (Hartman & Fauchald, 1971), an abyssal species from the Northwestern Atlantic synonymized by Pettibone (1992) with *Pholoides dorsipapillatus* (von Marenzeller, 1893), a species from the Mediterranean Sea. Within the scaleless sigalionids or Pisionins, five species have been recorded for this region, three were described from the Tropical Northwestern Atlantic, and two are questionable records. *Pisione hartmannschroederae* Westheide, 1995 was described from Florida; *P. wolfi* San Martín, López & Núñez, 1999 was described from Cuba; and *Pisionidens ixazaluohae* Petersen et al., 2016 was described from Quintana Roo, Mexico. Questionable records correspond to *Pisionidens indica* (Aiyar & Alikunhi, 1940) described from India and repeatedly recorded in the region (Dexter, 1974; Harper et al., 1979; Fauchald, 1973; Báez & Ardila, 2003), and *Pisione remota* (Southern, 1914) described from Ireland and recorded in Cuba (Ibarzábal, 1997, 2004). Unfortunately, these latter records do not include enough morphological evidence to confirm or deny the identity of the species.

In summary, in the region the study of these subfamilies has been intermittent or even neglected for long periods. In that context, this contribution aims to improve the knowledge of these subfamilies in the Tropical Northwestern Atlantic. Herein, two species of *Sthenelanella*, and one of *Taylorpholoe* are described as new, and *Pisione wolfi* is recorded from Southern Caribbean.

## Materials and Methods

The study area considers the Gulf of Mexico and the Caribbean Sea, two regions frequently referred together as the Grand Caribbean Region, covering from Bermuda to central Brazil (Salazar-Vallejo, 1996). However, since the area encompasses a wide range of geographic and oceanographic conditions that cannot be restricted to mention solely as Caribbean conditions; the revised classification of the marine regions and environments proposed by Spalding et al. (2007) was used in this contribution.

The examined material came from two Mexican scientific collections: the Reference Collection of Laboratorio de Biodiversidad y Cambio Climático (BIOMARCCA) and the Reference Collection of Benthos (ECOSUR) of El Colegio de la Frontera Sur. The specimens from BIOMARCCA were deposited in the ECOSUR collection, as it guarantees their safety and availability for further consultation. Benthic marine sigalionids were collected from several localities along the Tropical Northwestern Atlantic. Specimens were fixed using a 10% formalin-seawater solution and preserved in 70% ethanol. Examined specimens were temporally stained with Shirlastain-A to enhance morphological details, mainly prostomial and parapodial features. Once the specimens were stained, they were observed and photographed. For scaled sigalionids, elytra were dissected and cleaned immersed in a 1:1 white vinegar-ethanol solution. Elytra and parapodia were dissected from the right body side and mounted to be observed in anterior position in semi-permanent microscope preparations in a 1:1 ethanol-glycerol solution. Several photographs were taken in different focal planes using a Canon EOS REBEL T6 camera (Canon, Tokyo, Japan) mounted on a light microscope; the photographs were then stacked using HeliconFocus ver. 6.7.1 (HeliconSoft Limited, 2007). Furthermore, drawings of prostomia, parapodia, and elytra were made in Photoshop CC using a drawing tablet (Wacom Intuos Draw, Wacom, Saitama, Japan).

Some complete specimens were prepared for scanning electron microscopy (SEM). These were dehydrated in a series of different concentrations of alcohol and hexamethyldisilazane (HMDS). After air-dried, the specimens were mounted in aluminum stubs, coated with gold and observed using a JEOL-JSM-601Plus-LA scanning electron microscope at the Scanning Electron Microscopy Laboratory (LMEB), ECOSUR-Chetumal. Morphological terms follow Pettibone (1992), Aungtonya & Eibye-Jacobsen (2013) and Tilic et al. (2021). Through the text, acicular terminology follows Salazar-Vallejo (2020) by using the more euphonic alternatives notacicula and neuracicula to refer notoacicula and neuroacicula. Recently, Cruz-Gómez (2022) proposed a new classification for studying the neurochaetae in sigalionids and used it for the subfamily Pelogeniinae; here, this classification is followed for the subfamilies Sthenelanellinae and Pholoinae. The results are presented by subfamily following the order used in Gonzalez et al. (2018).

The electronic version of this article in Portable Document Format (PDF) will represent a published work according to the International Commission on Zoological Nomenclature (ICZN), and hence the new names contained in the electronic version are effectively published under that Code from the electronic edition alone. This published work and the nomenclatural acts it contains have been registered in ZooBank, the online registration system for the ICZN. The ZooBank LSIDs (Life Science Identifiers) can be resolved and the associated information viewed through any standard web browser by appending the LSID to the prefix http://zoobank.org/. The LSID for this publication is: urn:lsid:zoobank.org:pub:D8DB22A2-E239-4CAD-A34C-B3B12B17E1B9. The online version of this work is archived and available from the following digital repositories: PeerJ, PubMed Central SCIE and CLOCKSS.

## Results

### Systematics

**Subfamily Sthenelanellinae Gonzalez et al., 2018**

***Sthenelanella* Moore, 1910**

***Sthenelanella* Moore, 1910**: 391.

***Euleanira* Horst, 1916**: 12 (syn.).

**Type species.** *Sthenelanella uniformis* Moore, 1910 by monotypy.

**Diagnosis.** Sthenelanellins with prostomium rounded, as long as wide. Without facial tubercle. Median antennal ceratophore with semispherical, nodular auricles. Might lack palpal sheaths. Segment 1 might possess inner tentacular lobes (lamellae). Ctenidial pads present; lacking stylodes. Notopodia on median and posterior segments with large, specialized sacs that produce feltage chaetae used to reinforce tube. Notochaetae of two kinds, long verticillate or short geniculate. Neurochaetae spinigers with short, sickle- or rod-shaped blades, except on the first segments which are falcigers with articulate blades. Elytra might possess patterns of pigmentation (after Pettibone, 1969; Aungtonya & Eibye-Jacobsen, 2013; Tilic et al., 2021; Eibye-Jacobsen, Aungtonya & Gonzalez, 2022).

### Key to species of *Sthenelanella* Moore, 1910

**1** Tentacular segment without dorsal tentacular crest; posterior elytra with entire lateral margin **2**

- Tentacular segment with dorsal tentacular crest; posterior elytra with deeply sinuous lateral margin ***S. ehlersi*** **(Horst, 1916)**, Madura Strait, Java Sea
**2 (1)** Neurochaetae from anterior segments with articulate blades **4**

- Neurochaetae from anterior segment with entire blades **3**

**3 (2)** Elytral surface with mottled pigmentation; elytra on all segments from segment 27 ***S. uniformis* Moore, 1910**, California, Eastern Pacific
- Elytral surface transparent without pigmentation; elytra on all segments from segment 25 ***S. eylathae* (Fauvel, 1958)**, Gulf of Aqaba
**4 (2)** Branchiae from segment 2 **5**

- Branchiae from segment 5 ***S. petersini* Lana, 1991**, Brazil, Southwestern Atlantic
**5 (4)** Between segments 2 and 3 with dorsal ctendial pads **6**

- Between segments 2 and 3 without dorsal ctenidial pads ***S. corallicola* Thomassin, 1972**, Madagascar, Indian Ocean
**6 (5)** Posterior elytra with or without marginal papillae; anterior segments with neurochaetal blades with bifurcate tips **7**

- Posterior elytra with smooth margins; anterior segments with neurochaetal blades with tips entire ***Sthenelanella* sp. 1 Aungtonya & Eibye-Jacobsen, 2013**, Thailand, Andaman Sea
**7 (6)** Without inner palpal sheaths, elytral pigmentation pattern goldish transversally banded; posterior elytra without marginal fringes, **8**

- Inner palpal sheaths present, elytral pigmentation pattern brown mottled; posterior elytra with marginal short papillae ***S. japonica* Imajima, 2003**, Sagami Bay, Northwest Pacific
**8 (7)** Auricles small, 1/2 as large as median antenna ceratophore; first elytra rounded; elytral pigmentation transversally banded; posterior elytra reniform ***S. pechi* sp. nov.** Yucatan Peninsula, Southern Gulf of Mexico
- Auricles large, as long as median antenna ceratophore; first elytra subtriangular; elytral pigmentation mottled; posterior elytra rounded ***S. sarae* sp. nov.** Yucatan Peninsula, Western Caribbean

**Figure 1:**
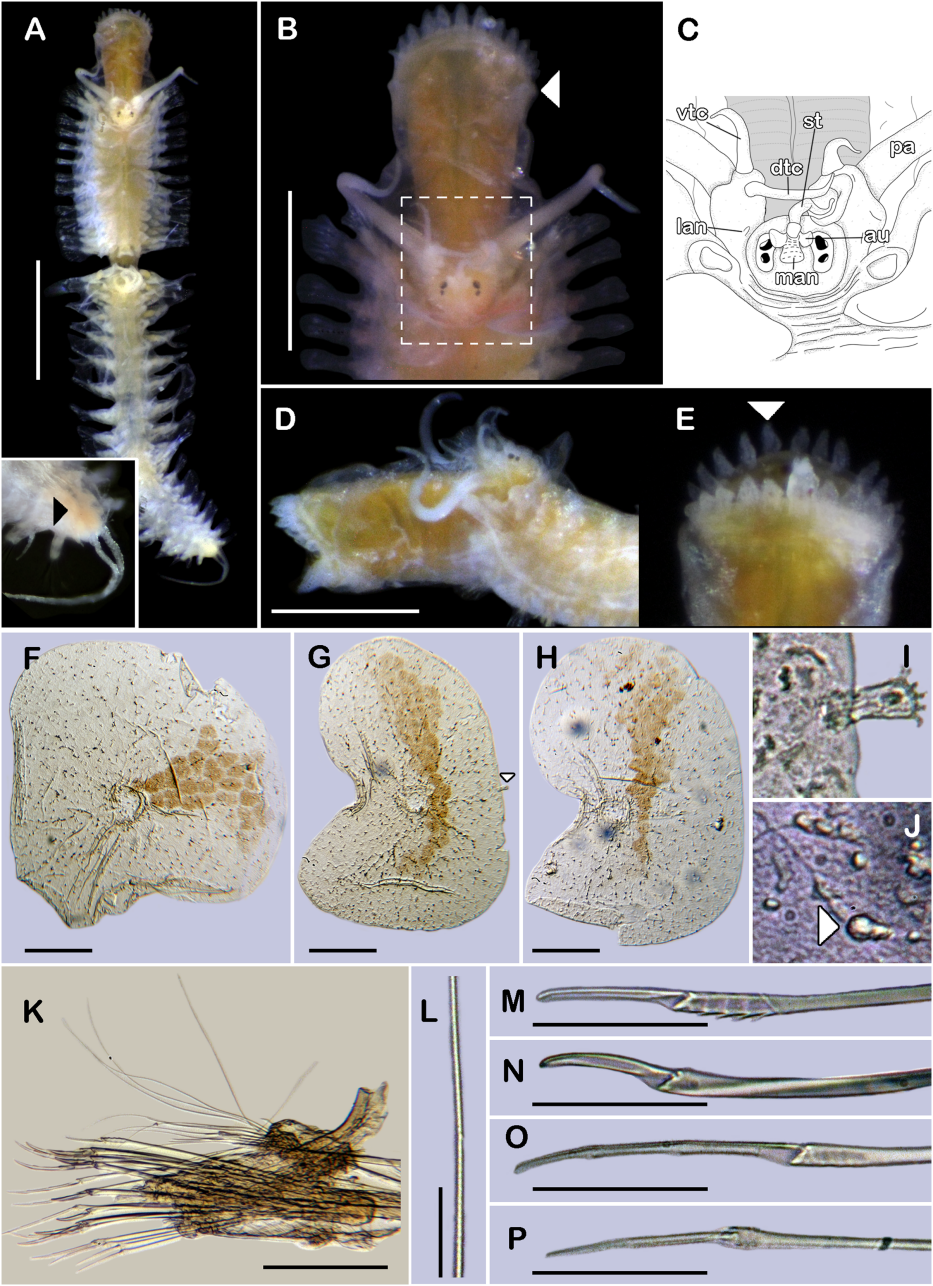
Holotype of *Sthenelanella pechi* sp. nov. (ECOSUR 293). (A) Complete specimen, dorsal view (insert: close-up pygidium, arrowhead indicates anus). (B) Anterior end, dorsal view, pharynx everted; arrowhead indicates lateral papillae. (C) Close-up of prostomium (chaetae omitted). (D) Anterior end, left lateral view. (E) Close-up of distal papillae, dorsal view; arrowhead indicates lanceolate papilla. (F) First right elytron. (G) Second right elytron. (H) Posterior right elytron. (I) Marginal papilla from F. (J) Superficial papillae from G. (K) Right parapodium from segment 2. (L) Notochaeta from same. (M) Unit A. (N) Unit B. (O) Unit C. (P) Unit D. Abbreviations: au, auricle; dtc, dorsal tentacular cirrus; lan, lateral antenna; pa, palp; st, style; vtc, ventral tentacular cirrus. Scale bars: A: 1 mm, B and D: 500 µm, F–H and K: 100 µm, M–P: 50 µm, L: 10 µm.

**Figure 2:**
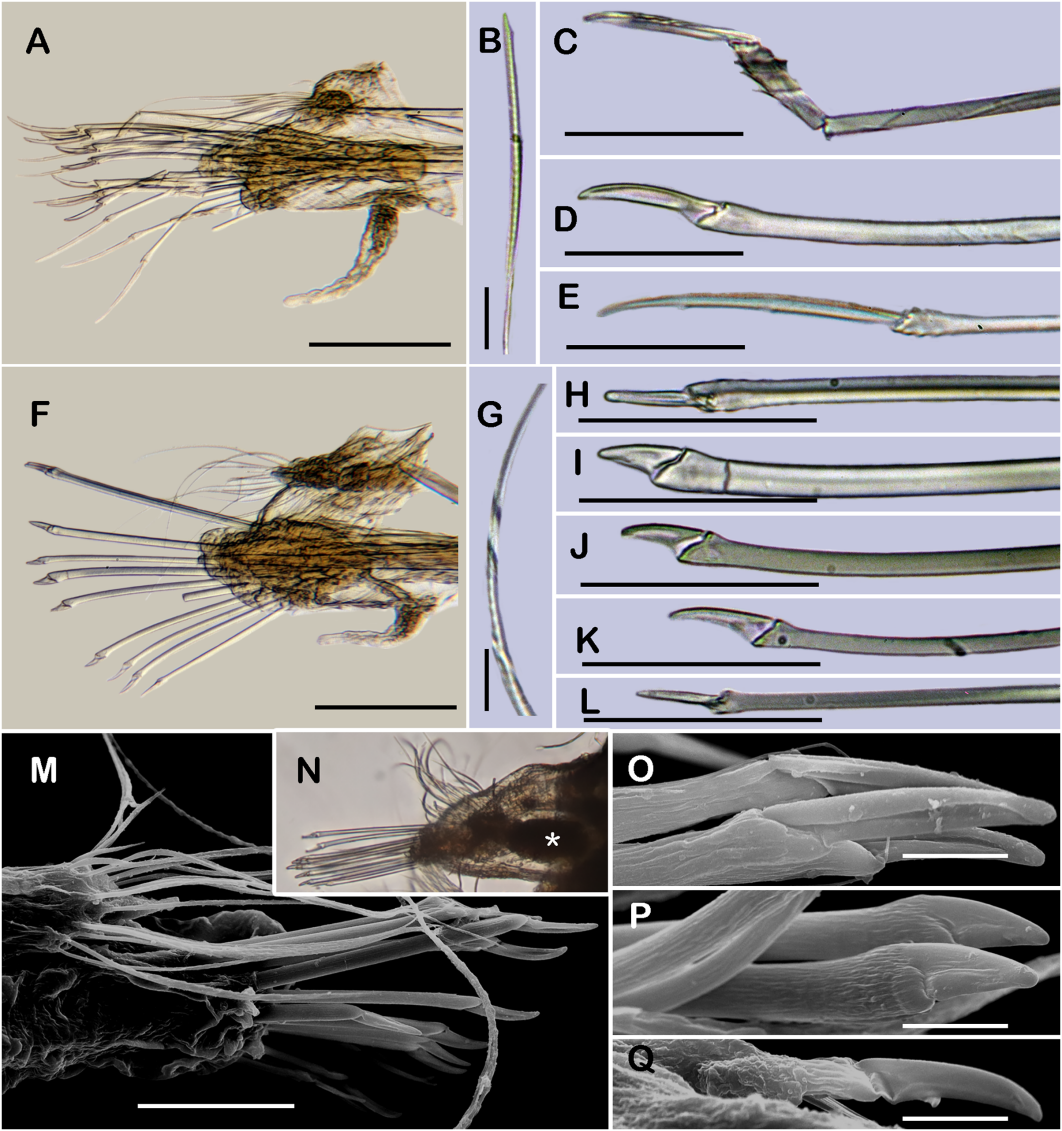
*Sthenelanella pechi* sp. nov. (A–L, N, holotype, ECOSUR 293; M–Q, paratype, ECOSUR 294). (A) Right parapodium from segment 3. (B) Notochaeta from same. (C) Unit A. (D) Unit B. (E) Unit C. (F) Right parapodium from segment 14. (G) Notochaeta from same. (H) Unit A. (I) Unit B. (J) Subunit 1. (K) Unit C. (L) Unit D. (M) SEM micrograph of right parapodium from segment 17. (N) Right parapodium from segment 16; asterisk indicates chaetal sac. (M) Unit A. (P) Unit B. (Q) Unit D. Scale bars: A and F: 100 µm, C–E and H–M: 50 µm, B, G, M and O–Q: 10 µm. Photo credit of M and O–Q: Sergio I. Salazar-Vallejo.

**Figure 3:**
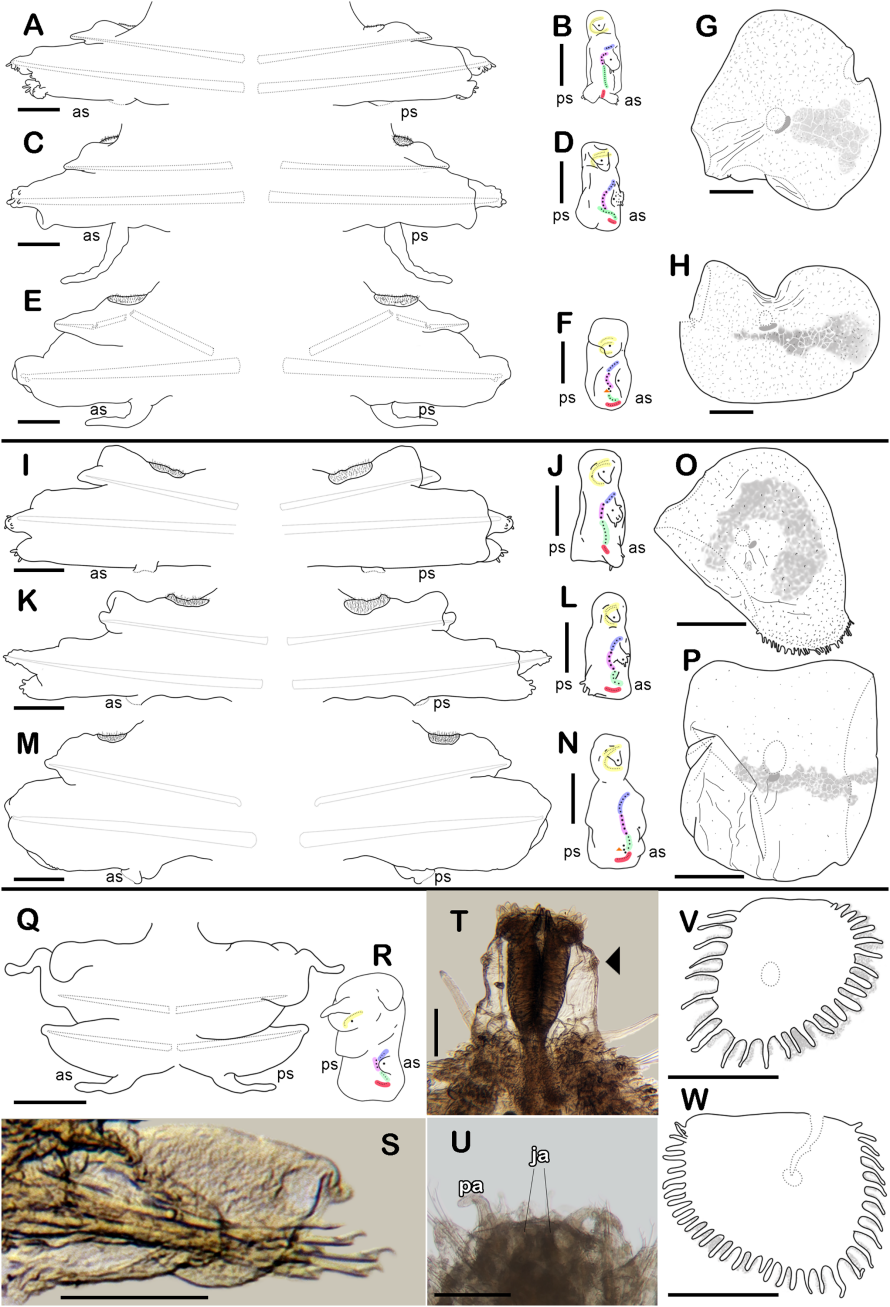
Holotype of *Sthenelanella pechi* sp. nov. (A–H), holotype of *Sthenelanella sarae* sp. nov. (I–N), *Taylorpholoe anabelae* sp. nov. ((Q–S, U–W) holotype; (T) paratype, ECOSUR 297). *Sthenelanella pechi* sp. nov. (A) Drawings of parapodium from segment 2. (B) Lateral view. (C) Drawings of parapodium from segment 3. (D) Lateral view. (E) Drawings of parapodium from segment 14. (F) Lateral view. (G) Drawing of first right elytron. (H) Drawing of posterior right elytron. *Sthenelanella sarae* sp. nov. (I) Drawings of parapodium from segment 2. (J) Lateral view. (K) Drawings of parapodium from segment 3. (L) Lateral view. (M) Drawings of parapodium from segment 18. (N) Lateral view. (O) Drawing of first right elytron. (P) Drawing of posterior right elytron. *Taylorpholoe anabelae* sp. nov. (Q) Drawings of parapodium from segment 8. (R) Lateral view. (S) Parapodium from segment 8, ventral view. (T) Paratype, pharynx everted; arrowhead indicates lateral papillae. (U) Holotype, anterior end, ventral view. (V) Drawing of second right elytrum. (W) Drawing of posterior right elytrum. Abbreviations: as, anterior side; ja, jaws; ps, posterior side. Scale bars: O–P: 200 µm, G–N, T–W: 100 µm, A–F and Q–S: 50 µm.

***Sthenelanella*** ***pechi* sp. nov.**

(Figures 1, 2 and 3A–3H)

**Type material.** Holotype (ECOSUR 293): Southern Gulf of Mexico, Veracruz, off Tecolutla (20°46′42.30″N, 96°58′26.58″W); O/V Justo Sierra; 49 m; fine sand; March 17, 2018; collected by Anabel León. Paratype. One specimen (ECOSUR 294); same data as holotype; (coated in gold, fragment poorly fixed without posterior region, 15 segments, 0.35 cm long, 0.1 cm wide).

**Description of holotype (ECOSUR 293).** Specimen complete with 32 segments, 0.5 cm long, 0.42 cm to segment 30, 0.1 cm wide. Body pale orange, translucent posteriorly, short, broad (Fig. 1A). Mid-dorsal line smooth, some elytra lost, venter smooth. Elytra on segments 2, 4, 5, 7, alternate segments to 25, then present in all segments. Elytrophores bulbous, short, slightly larger in posterior segments.

Prostomium pale orange, oval, wider than long. Two pairs of eyes, anterior eyes larger, visible dorsally. Lateral antennae short, barely seen, dorsally fused with tentacular segment. Median antenna with short ceratophore, 1/3 as long as prostomial length; basally with two auricles semispherical with margins bent, small, 1/2 as large as median antenna ceratophore; style short, twice as long as ceratophore (Figs. 1B and 1C). Without dorsal tentacular crest. First segment uniramous, chaetae simple verticillate. Dorsal tentacular cirri long, three times longer than neuropodia, ventral cirri shorter than dorsal tentacular cirri. Palps long, reaching segment nine, without palp sheaths. (Fig. 1B). Pharynx everted with 24 distal lanceolate papillae (Figs. 1B, 1D and 1E), and two subdistal lateral papillae (Fig. 1B, arrow). Ctenidial pads from segment three, three pads: one placed below elytrophores, other inserted dorsolaterally above the notopodia, and other on the dorsal side of the notopodia. Branchiae from segment two, placed below the elytrophore or tubercles, conical, short, 1/2 as large as elytrophores, cilia not perceived.

First right elytron small, rounded, fragile (Figs. 1F and 3G). Second right elytron larger, oblong, notched (Fig. 1G), a filiform single marginal papilla with four sensory hairs (Fig. 1I). Posterior elytra larger, oblong, notched, with smooth margins (Figs. 1H and 3H). Surface of all elytra with golden spots as transversal band pattern, and scattered homogenously tiny globular papillae (Fig. 1J).

Right parapodium from segment 2, biramous (Figs. 1K, 3A and 3B). Notopodia truncated, smooth, short, 1/3 as long as neuropodia; notacicular lobe as long as notopodia. With up to 15 simple verticillate notochaetae, shortest 1/4 as long as notopodia; longest six times as long (Fig. 1L). Neuropodia conical, smooth, lateral margins with a few filiform papillae; neuracicular lobe truncate, distally with short filiform papillae, acicula blunt, translucent, penetrating in the distal section of the lobe (Figs. 1K and 3A). Neurochaetae only falcigers: unit A (upper group), one falciger with handle thick with seven transverse rows of spines on its sides, blades medium-sized, eight times as long as wide, (Fig. 1M); unit B (median group), five falcigers with handles thick smooth, blades medium-sized, 7–8 times as long as wide (Fig. 1N); unit C (lower group), 10 falcigers with handles slender with 4–5 transverse rows of spines on its sides, blades articulate, long, 15–17 times as long as wide, (Fig. 1O); unit D (lowest group), four falcigers with slender handle with four transverse rows of spines on its sides, blades articulate, long, 11–13 times as long as wide (Fig. 1P). Ventral cirri long, barely longer than neuropodia, lost during preparation of the slide. Buccal aperture wide, anteroventrally inserted.

Right parapodium from segment 3, biramous (Figs. 2A, 3C and 3D). Notopodia truncated, smooth, short, 1/3 as long as neuropodia; notacicular lobe short, 1/2 as long as notopodia. With up to 10 simple verticillate notochaetae, shortest as long as notacicular lobe, longest, four times as long (Fig. 2B). Neuropodia conical, smooth, lateral margins with short filiform papillae; neuracicular lobe slightly bilobed, distally with short filiform papillae, acicula blunt, translucent, penetrating the upper lobe in the distal section of the lobe (Figs. 2A and 3C). Neurochaetae only falcigers: unit A (upper group), one falciger with handle (broken) thick with six transverse rows of spines on its sides, blade medium-sized, eight times as long as wide (Fig. 2C); unit B (median group), four falcigers with handles thick, smooth, blades medium-sized, 4–5 times as long as wide (Fig. 2D); unit C (lower group), four falcigers with handles slender with 3–4 transverse row of spines on its sides, blades articulate, long, 13–15 times as long as wide (Fig. 2E); unit D (lowest group), only two slender handles remain. Ventral cirri, long, barely longer than neuropodia.

Right parapodium from segment 14 (middle segment), biramous (Figs. 2F, 2M, 3E and 3F). Notopodia conical, really reduced, smooth, 1/4 as long as neuropodia. Chaetal sac present from this segment and posterior (Fig. 2N), small, content in the notopodia, lost during the preparation of the slide. With up to 10 simple verticillate notochaetae, shortest as long as notopodia, longest five times as long (Fig. 2G). Neuropodia conical, margins with reduced papillae. Neuracicula translucent, distal end obliquely swollen, subdistal tooth smaller (Figs. 2F and 3E). Neurochaetae only spinigers: unit A (upper group), two spinigers with handles thick with three rows of spines, blades medium-sized, four times longer than wide (Figs. 2H and 2O); unit B (median group), four spinigers with handles thick smooth, blades short, two times longer than wide, (Figs. 2I and 2P); unit C (lower group), one spiniger with handle thick, smooth, blade medium-sized (Fig. 2J); subunit 1 (between units C and D), two spinigers with handles thick smooth, blades medium-sized, 3–4 times longer than wide (Fig. 2K); unit D (lowest group), one spiniger with handle slender smooth, blade medium-sized (Figs. 2L and 2Q). Ventral cirri, short, 1/2 as long as neuropodia.

Posterior region tapered, pygidium depressed, rounded, and glandular with two long anal cirri, as long as the last six segments, anus dorso-terminal (Fig. 1A, insert).

**Type locality.** Off Tecolutla (20°46′42.30″N, 96°58′26.58″W), Veracruz, Mexico.

**Distribution.** Tropical Northwestern Atlantic: Southern Gulf of Mexico, only known from the type locality.

**Etymology.** The species is named after Daniel Pech (ECOSUR-Campeche) in recognition of his efforts in studying marine benthos in the Gulf of Mexico. Also, he kindly made available most of the material examined in this study. The epithet is a noun in the genitive case (ICZN, 1999, Art. 31.1.2).

**Remarks.** The genus *Sthenelanella* has been recorded from warm temperate and tropical waters, mostly from the Indo-Pacific coasts. From the Western Pacific, *S. japonica* Imajima, 2003 agrees with *S. pechi* in some features, such as lacking a dorsal tentacular crest and having a papillate neuracicular lobe. However, *S. japonica* stands out among *Sthenelanella* in having inner palpal sheaths in the basis of the palps, whereas *S. pechi* sp. nov. lacks palpal sheaths. Another difference is the pigmentation pattern in the posterior elytra. *Sthenelanella pechi* sp. nov. has elytra with a mottled band pattern, occurring from the elytral insertion to the lateral margin of the elytra, where the pattern tends to be sparse, and the color gets lighter, whereas *S. japonica* has elytra with a mottled random pattern, with spots occurring near to the elytral insertion, and in the lateral and proximal margin of the elytra, with its color uniform. Another species that resembles to *S. pechi* sp. nov. is *S. corallicola* Thomassin, 1972 from Madagascar; both species possess the first elytra rounded and blades articulate in neurochaetae from anterior segments. However, *S. pechi* sp. nov. differs from *S. carallicola* in having ctenidial pads from segment two, elytra from posterior segments oblong with smooth margins, and a mottled band pattern. Whereas *S. corallicola* has ctenidial pads from segment three, elytra from posterior segments subreniform with submarginal papillae in lateral borders, and colorless. Another difference between these two species is the number of pharyngeal papillae. The holotype of *S. pechi* sp. nov. shows the pharynx completely everted with 12 paired papillae, 24 papillae in total, while *S. corallicola* shows about 14 paired papillae, 28 papillae in total (Thomassin, 1972: 256, figs. a, c).

From the Southwestern Atlantic, only one species of *Sthenelanella* has been described: *S. peterseni* Lana, 1991. The newly described *Sthenelanella pechi* sp. nov. resembles *S. peterseni* in having posterior elytra with smooth entire margins, articulate blades in neurochaetae from anterior segments, and neuracicular lobe with papillae. However, *S. pechi* sp. nov. differs from *S. peterseni* in having larger auricles, 1/2 as long as the median antenna ceratophore, neurochaetae from anterior segments with entire blades, and branchiae from segment two. Whereas *S. peterseni* has small auricles, 1/4 as long as its median antenna ceratophore, neurochaetae from anterior segments with entire or bifid blades, and branchiae from segment five. Another species in the region that resembles *S. pechi* sp. nov. is *S. sarae* sp. nov., described below; however, obvious differences can be found in prostomial and elytral features. *Sthenelanella pechi* sp. nov. has small auricles, 1/2 as long as the median antenna ceratophore, first elytra rounded with smooth margins, with mottled semi band pattern occurring from the elytral insertion to the lateral margin, posterior elytra oblong with mottled band pattern. Whereas *S. sarae* sp. nov. has large auricles, as long as the median antenna ceratophore, first elytra rounded with several lateral papillae, with mottled “C” shaped pattern, surrounding the elytral insertion, posterior elytra rounded with a narrower mottled band pattern. Despite these two species are found in the same region, they are associated with different bathymetric distributions, *S. pechi* sp. nov. is distributed in shallow waters, less than 50 m deep, while *S. sarae* sp. nov. is found in deeper waters, between 76–148 m deep.

**Figure 4:**
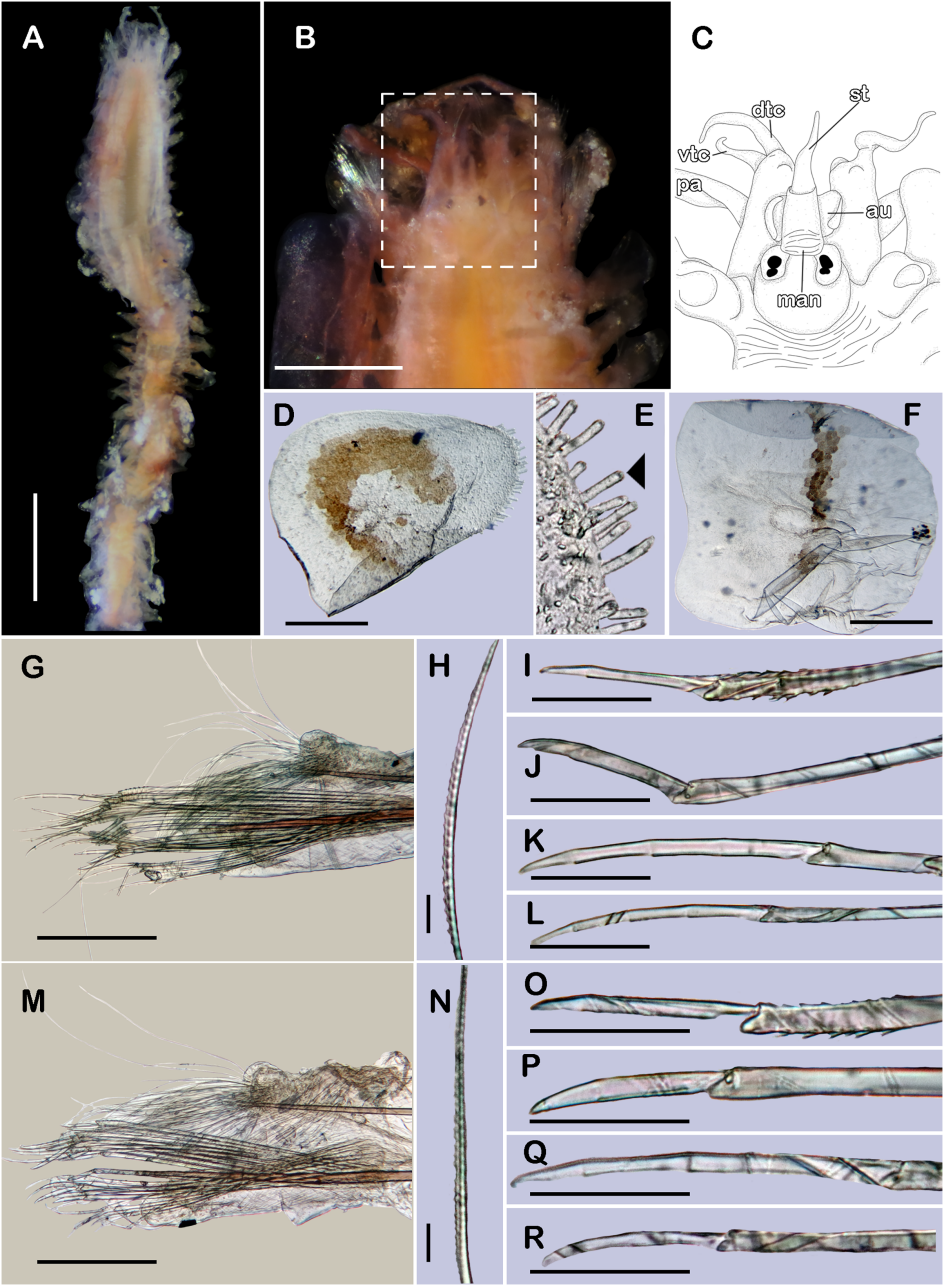
Holotype of *Sthenelanella sarae* sp. nov. (ECOSUR 295). (A) Incomplete specimen, dorsal view. (B) Anterior end, dorsal view, pharynx partially everted. (C) Close-up of prostomium (chaetae omitted). (D) First right elytron. (E) Margin of E; arrowhead indicates marginal papilla. (F) Posterior right elytron. (G) Right parapodium from segment 2. (H) Notochaeta from same. (I) Unit A. (J) Unit B. (K) Unit C. (L) Unit D. (M) Right parapodium from segment 3. (N) Notochaeta from same. (O) Unit A. (P) Unit B. (Q) Unit C. (R) Unit D. Abbreviations: au, auricle; dtc, dorsal tentacular cirrus; pa, palp; vtc, ventral tentacular cirrus. Scale bars: A: 2 mm, B: 500 µm, D, F, G and M: 200 µm, I–L and O–R: 50 µm, H and N: 10 µm.

**Figure 5:**
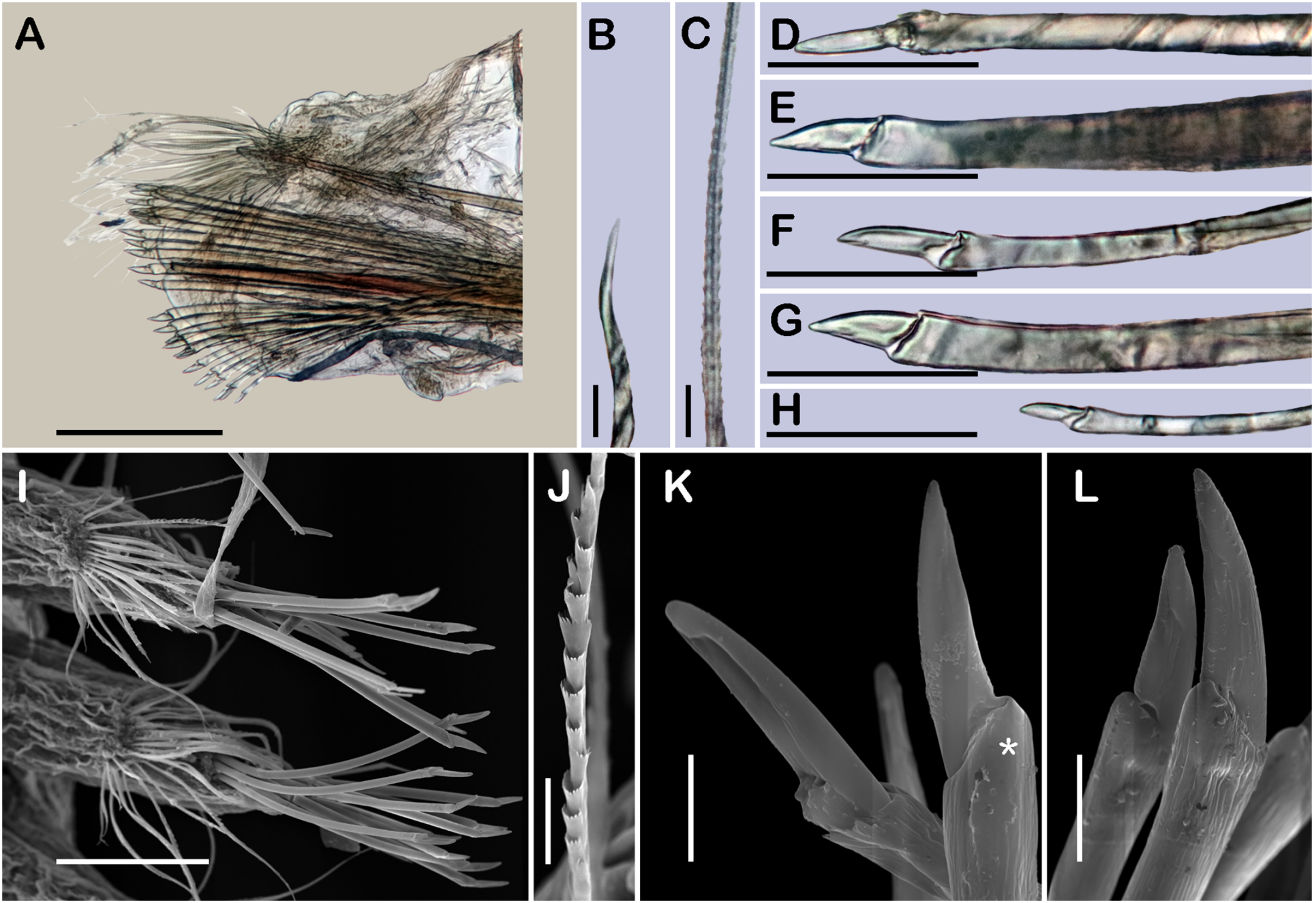
*Sthenelanella sarae* sp. nov. ((A–H) holotype, ECOSUR 295; (I–L) non-type, ECOSUR P3232). (A) Right parapodium from segment 18. (B) Geniculate notochaeta from same. (C) Verticillate notochaetae from same. (D) Unit A. (E) Unit B. (F) Unit C. (G) Subunit 1. (H) Unit D. (I) SEM micrographs of right parapodium from segments 13 and 14. (J) Notochaeta from same. (K) Units A and B; asterisk indicates unit B. (L) Unit C. Scale bars: A: 200 µm, I: 100 µm, D–H: 50 µm, B, C and J–L: 10 µm. Photo credit of I–L: Sergio I. Salazar-Vallejo.

***Sthenelanella sarae* sp. nov.**

(Figures 3I–3P, 4 and 5)

*?Sthenelanella* sp. A Wolf, 1984: 25-23, fig. 25-20.

**Type material.** Holotype (ECOSUR 295): Western Caribbean, off NE Yucatan Peninsula (23°18′0″N, 87°37′0″W); O/V Justo Sierra; 148 m; silt; September 19, 2010; collected by Anabel León.

**Additional material**

**Mexico, Veracruz.** One specimen (ECOSUR P3232); off Tecolutla (20°43′8.10″N, 96°51′11.09″W); O/V Justo Sierra; 76 m; fine sand; March 17, 2018; collected by Anabel León (coated in gold, complete, poorly fixed 50 segments, 0.8 cm long, 0.5 cm to segment 30, 0.1 cm wide).

**Description of holotype (ECOSUR 295).** Specimen incomplete with 21 segments, 1.2 cm long, 0.2 cm wide. Body translucent, damaged posteriorly, broad (Fig. 4A). Mid-dorsal line smooth, some elytra lost, venter smooth. Elytra on segments 2, 4, 5, 7, alternate segments to 25, then present in all segments. Elytrophores flatten, short, slightly larger in posterior segments.

Prostomium whitish, oval, wider than long. Two pairs of eyes, anterior eyes larger, all eyes visible dorsally. Lateral antennae short, barely seen, dorsally fused with tentacular segment. Median antenna with long ceratophore, as long as prostomial length; basally with two auricles ear-shaped with margins bent, large, as long as median antenna ceratophore; style short, twice as long as ceratophore (Fig. 4C). Without dorsal tentacular crest. First segment uniramous, chaetae simple verticillate. Dorsal tentacular cirri short, as long as neuropodia, ventral cirri shorter than dorsal tentacular cirri. Palps long, reaching segment 8, without palp sheaths (Fig. 4B). Pharynx partially everted, ECOSUR P3232 specimen with pharynx damaged, fully everted with distal 28 lanceolate papillae and two subdistal lateral papillae. Ctenidial pads from segment three, three pads: one placed below elytrophores, other inserted dorsolaterally above the notopodia, and other on the dorsal side of the notopodia. Branchiae from segment 2, placed below the elytrophore or tubercle, flat and tapered, short, 1/4 as large as elytrophores, cilia not perceived.

First right elytron small, rounded, fragile, with several short filiform marginal papillae (Figs. 3O, 4D and 4E). Second right elytron missing. Posterior right elytra larger, rounded, with smooth margins (Figs. 3P and 4F). Surface of all elytra partially colored with brown spots as mottled “C” shaped pattern in the first pair of elytra, in posterior elytra with mottled band pattern, all elytra with few small globular papillae (Fig. 4F).

Right parapodium from segment 2, biramous (Figs. 3I, 3J and 4G). Notopodia truncated, smooth, short, 1/3 as long as neuropodia; notacicular lobe long, 1/2 as long as notopodia. With up to 30 simple verticillate notochaetae, shortest twice as long as notopodia; longest eight times as long (Fig. 4H). Neuropodia conical, with a large non-acicular lobe ventrally, lateral margins with a few filiform papillae; neuracicular lobe truncate, distally with short filiform papillae, acicula blunt, reddish, penetrating in the distal section of the lobe (Figs. 3I and 4G). Neurochaetae only falcigers: unit A (upper group), three falcigers with handles thick with nine transverse rows of spines on their sides, blades medium-sized, eight times as long as wide, (Fig. 4I); unit B (median group), five falcigers with handles thick smooth, blades medium-sized, 5–6 times as long as wide, (Fig. 4J); unit C (lower group), six falcigers with handles thick smooth, blades articulate, long, 13–15 times as long as wide (Fig. 4K); unit D (lowest group), five falcigers with slender handles with five transverse rows of spines on their sides, blades articulate, long, 11–13 times as long as wide (Fig. 4L). Ventral cirri long, twice as long as neuropodia. Buccal aperture wide, anteroventrally inserted.

Right parapodium from segment 3, biramous (Figs. 3K, 3L and 4M). Notopodia rounded, smooth, short, 1/3 as long as neuropodia; notacicular lobe short, as long as notopodia. With up to 30 simple verticillate notochaetae, shortest twice as long as notopodia, longest seven times as long (Fig. 4N). Neuropodia conical, smooth, with a non-acicular lobe ventrally, lateral margins with a few short filiform papillae; neuracicular lobe massive, truncate, distally with short filiform papillae, acicula blunt, reddish, penetrating in the subdistal section of the lobe (Figs. 3K and 4M). Neurochaetae only falcigers: unit A (upper group), three falcigers with handles thick with six transverse rows of spines on their sides, blades medium-sized, nine times as long as wide (Fig. 4O); unit B (median group), seven falcigers with handles thick, smooth, blades medium-sized, 7–9 times as long as wide (Fig. 4P); unit C (lower group), four falcigers with handles slender smooth, blades articulate, long, 13–14 times as long as wide (Fig. 4Q); unit D (lowest group), five falcigers with handles slender with four rows of spines on its sides, blades articulate, long, 14–15 times as long as wide (Fig. 4R). Ventral cirri short, slightly shorter than neuropodia.

Right parapodium from segment 18 (middle segment), biramous (Figs. 3M, 3N, 5A and 5I). Notopodia rounded, short, smooth, 1/2 as long as neuropodia, notacicular lobe short. Chaetal sac large, content in the notopodia and partially in neuropodia, lost during preparation of the slide, present from segment 14. Notochaetae of two kinds, seven simple thick geniculate ones, short, 1/4 as long as notopodia (Fig. 5B), and up to 20 simple verticillate notochaetae, shortest 1/2 as long as notopodia, longest twice as long as notopodia (Figs. 5C and 5J). Neuropodia truncated, margins with reduced papillae. Neuracicula translucent, distal end obliquely barely swollen, subdistal tooth as long as distal one (Figs. 3M and 5A). Neurochaetae only spinigers: unit A (upper group), five spinigers with handles thick with three rows of spines on its sides, blades medium-sized, three times as long as wide (Figs. 5D and 5K); unit B (median group), four spinigers with handles thick smooth, blades short, twice as long as wide (Figs. 5E and 5K, asterisk); unit C (lower group), four spinigers with handles thick smooth, blades medium-sized, three times as long as wide (Figs. 5F and 5L); subunit 1 (between units C and D), two spinigers with handles thick and smooth, blades short, twice as long as wide (Fig. 5G), unit D (lowest group), two spinigers with handles slender smooth, blades medium-sized, three times as long as wide (Fig. 5H). Ventral cirri short, 1/2 as long as neuropodia.

Posterior region lost.

**Type locality.** Off Northeastern Yucatan Peninsula (23°18′0″N, 87°37′0″W), Southern Gulf of Mexico.

**Distribution.** Tropical Northwestern Atlantic: Floridian ecoregion, Southern Gulf of Mexico and Western Caribbean; from Florida, USA to Yucatan Peninsula, Mexico (Wolf, 1984).

**Etymology.** The species is named after Sara Balam, in recognition of her effort during many years as a technician assistant in the Laboratorio de Biodiversidad y Cambio Climático, ECOSUR-Campeche. The epithet is a noun in the genitive case (ICZN, 1999, Art. 31.1.2).

**Remarks.** In the Gulf of Mexico, the only record of the genus *Sthenelanella* was made by Wolf (1984) from Florida and Alabama. He described and illustrated *Sthenelanella* sp. A, characterized by having large auricles, as long as the median antenna ceratophore (Wolf, 1984: 25-22, fig. 25-20a), first elytra rounded with several lateral papillae and with a mottled “C” shaped pattern near to the elytral insertion (Wolf, 1984: 25-22, fig. 25-20b, c), and blades with bifurcate tips in neurochaetae from anterior segments (Wolf, 1984: 25-22, fig. 25-20k, m–p). These features seem to agree with those found in *S. sarae* sp. nov., thus, it is possible that Wolf’s previous record refer to the newly described *S. sarae* sp. nov. The latter will be confirmed once Wolf’s specimens are studied.

Wolf (1984: 25-24) compared *Sthenelanella* sp. A with *S. ehlersi* (Horst, 1916) and *S. corallicola*, and stated some differences found in *Sthenelanella* sp. A, such as the bifid blades in neurochaetae from segments 2–5, the rounded and pigmented “C” shaped pattern in the first elytra pair, and the highly papillate neuropodial lobe in segment two. One additional difference is the large size of its auricles, being as long as the median antenna ceratophore, while in *S. ehlersi* and *S. corallicola* the auricles are short, both about 2/3 as long as the median antenna ceratophore (Thomassin, 1972: 256, figs. 1a, 1c; Pettibone, 1969: 435, fig. 1a).

*Sthenelanella sarae* sp. nov. also stands out by having neurochaetae with thick handles in posterior segments, the same width along the handle, including the joint with the blade. This kind of neurochaetae is also found in *S. uniformis* Moore, 1910, *S. japonica* and *S. peterseni*. However, *S. sarae* sp. nov. differs from *S. uniformis* in having posterior elytra rounded with a mottled transversal band, whereas *S. uniformis* has posterior elytra reniform with a large brown patch on the anterior elytral margins (Moore, 1910: Pl. 33, fig. 108; Pettibone, 1969: 432, fig. 2b). Regarding *S. japonica* and *S. peterseni*, *S. sarae* sp. nov. differs from both species in having particularly large auricles, as long as median antenna ceratophore, and posterior elytra with a mottled transversal band pattern, while both species possess small auricles, about 1/4 and 1/2 as large as median antenna ceratophore, respectively, and posterior elytra with an undefined mottled pattern or without pigmentation, respectively. On the other hand, *S. sarae* sp. nov. shares distribution in the Gulf of Mexico with another *Sthenelanella* species herein described, *S. pechi* sp. nov. Both species are easily differentiated based on the size of the auricles, the pigmentation pattern in the first elytra, and the shape and pigmentation pattern in the posterior elytra (see remarks section of *S. pechi* sp. nov.).

**Subfamily Pholoinae Kinberg, 1858**

***Taylorpholoe*** **Pettibone, 1992**

***Taylorpholoe*** **Pettibone, 1992**: 13

**Type species.** *Pholoe minuta hirsuta* Rullier & Amoureux, 1979 by original designation.

**Diagnosis.** Pholoins with scattered tubercles on the dorsal side of the body. Prostomium bilobed, rounded. Median antenna inserted occipitally on prostomium. Lateral antennae present. Facial tubercle present. Notochaetae short geniculate simple chaetae. Neurochaetae always falcigers with tapered curved blades. Embryos brooded beneath elytra. Interstitial animals (after Pettibone, 1992; Eibye-Jacobsen, Aungtonya & Gonzalez, 2022).

### Key to species of *Taylorpholoe* Pettibone, 1992

**1** Dorsal papillae small and flat, present only in the middorsal line; first elytra rounded with few long marginal papillae; basis of notopodial stylode small, it can be as wide as neuropodial lobe ***T. hirsuta* (Rullier & Amoureux, 1979)**, Brazil, Southwestern Atlantic.
- Dorsal papillae large and bulbous, covering all the dorsal surface; first elytra subtriangular with numerous short marginal papillae; basis of notopodial stylode massive, 2–3 times as wide as neuropodial lobe ***T. anabelae* sp. nov.**, Yucatan Peninsula, Western Caribbean.

**Figure 6:**
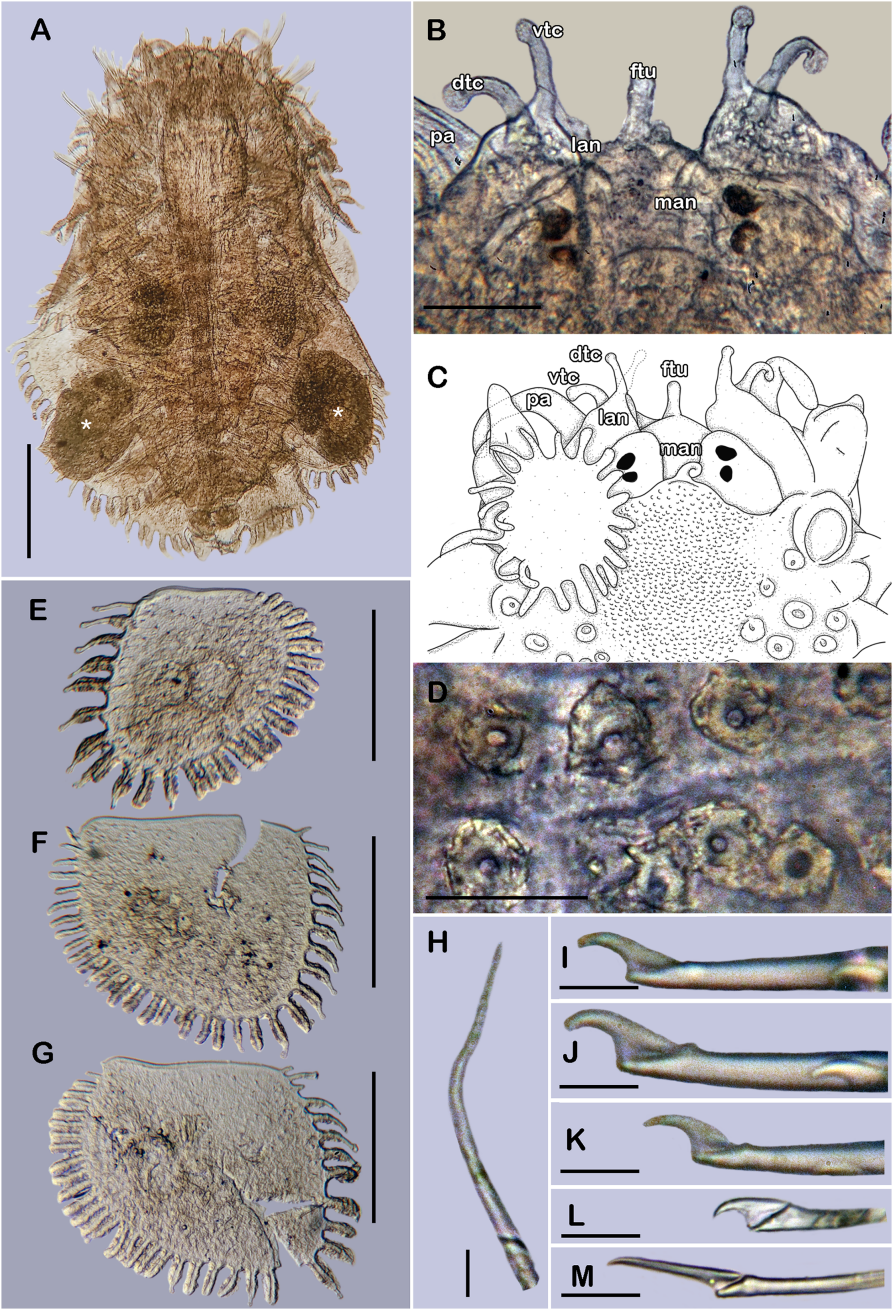
Holotype of *Taylorpholoe anabelae* sp. nov. (ECOSUR 296). (A) Complete specimen, dorsal view; asterisk indicated embryos in posterior elytra. (B) Close-up of prostomium. (C) Drawing of prostomium (chaetae omitted). (D) Dorsal papillae from middle segments. (E) Second right elytron. (F) Posterior right elytron. (G) Posterior right elytron with embryo. (H) Notochaeta from segment 8. (I) Unit A. (J) Unit B. (K) Unit C. (K) Unit D. (L) Spiniger from anterior segments. Abbreviations: dtc, tentacular cirrus; ftu, facial tubercle; lan, lateral antenna; man, median antenna; pa, palp; vdt, ventral cirrus. Sale bars: A: 200 µm, E–G: 100 µm, B: 50 µm, D: 25 µm, H–M: 10 µm.

**Figure 7:**
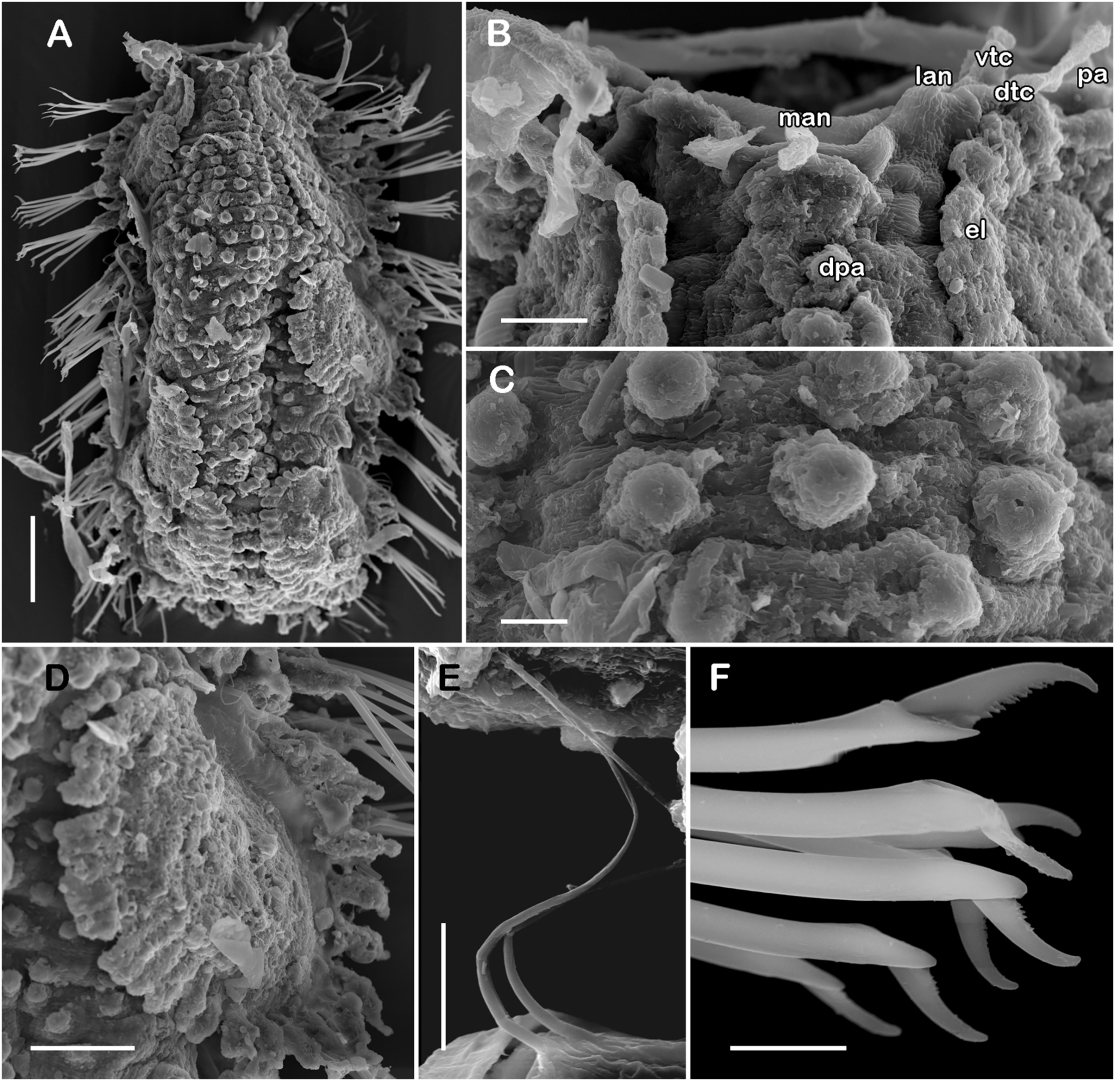
SEM micrographs of paratype of *Taylorpholoe anabelae* sp. nov. (ECOSUR 297). (A) Complete specimen. (B) Anterior end, dorsal view, prostomium partially folded. (C) Dorsal papillae from middle segments. (D) First right elytron. (E) Notochaeta from posterior segments. (F) Neurochaetae from E. Abbreviations: el, elytron; dpa, dorsal papilla; dtc, tentacular cirrus; man, median antenna; pa, palp; vtc, ventral tenticular cirrus. Scale bars: A, 100 µm, B: 20 µm, C–F: 10 µm. Photo credit: Sergio I. Salazar-Vallejo.

***Taylorpholoe anabelae* sp. nov.**

(Figures 3Q–3W, 6 and 7)

*?Pholoe* sp.—Taylor, 1971: 111, fig. 3E-1 (*non T. hirsuta* Rullier & Amoureux, 1979).

*?*Sigalionidae gen. sp.—San Martín, Aguirre & Baratech, 1986: 9, fig. 5.

*?Taylorpholoe hirsuta*.—Pettibone, 1992: 14, figs. C–M (*partim, non* Rullier & Amoureux, 1979).

**Type material.** Holotype (ECOSUR 296): Western Caribbean, Quintana Roo, off Holbox (22°9′59.28″N, 87°12′59.10″W); O/V Justo Sierra; 37 m; coarse sand; November 30, 2012; collected by Sara B. Balam. Paratype. 1 specimen (ECOSUR 297): off Holbox (22°4′0.22″N, 86°55′0.11″W); O/V Justo Sierra; 42 m; fine sand; November 29, 2012; collected by Anabel León, (coated with gold, complete, 15 segments, 1.1 mm long, 0.6 mm wide).

**Additional material**

**Mexico, Quintana Roo.** One specimen (ECOSUR P3233): Nichupté Lagoon (21°6′36.25″N, 86°46′59.07″W); July 06, 1988; seagrass; collected by Soledad Jiménez & José Oliva (14 segments, 1 mm long, 0.9 wide).

**Description of holotype (ECOSUR 296).** Specimen complete with 13 segments, 0.8 cm long, 0.6 cm wide. Female, oocytes internally visible; early juveniles beneath posterior elytra, segmentation not perceived, oval shaped, small, 213 µm length, 140 µm width. Body translucent, depressed, short, broad (Figs. 6A and 7A). Dorsal papillae, large bulbous, scattered on the dorsal surface, posterior to prostomium granular surface; ventral papillae, short and globular (Figs. 6C, 6D and 7C). Elytra on segments 2, 4, 5, 7 alternate segments to end. Elytrophores bulbous, short, as large as notopodia.

Prostomium partially fused with the first segment, oval, wider than long. Two pairs of eyes, all of similar size, visible dorsally. Lateral antennae short, inserted on the anterior border of the prostomium in the cephalic peak. Median antenna inserted occipitally on prostomium, short, 1/4 as long as prostomium (Figs. 6B, 6C and 7B). First segment directed anteriorly, achaetous, dorsal, and ventral tentacular cirri short. Palps short thick, laterally displaced, reaching segment two (Figs. 6A and 6C).

All elytra smooth, fringe with filiform papillae; first right elytron small, subtriangular, fimbria with 20 papillae (Figs. 6C and 7D). Second right elytron slightly larger than the first one, rounded, anterior margin smooth with 27 papillae (Figs. 3V and 6E). Posterior right elytra larger, rounded, fimbria with 34 marginal papillae, anterior margin smooth (Figs. 3W and 6F). Brooding elytra slightly larger, present in segment 10 (Fig. 6G).

Right parapodium from segment 8 (middle segment), biramous (Figs. 3Q and 3R). Notopodia foliaceous, large, smooth, twice longer than neuropodia, with a distal stylode basally broad. With up to 10 simple geniculate notochaetae, all with similar size, as long as notopodia (Figs. 6H and 7E). Neuropodia conical, short, surface scattered with tiny papillae. Neurochaetae only falcigers with short, curved blades (Fig. 7F): unit A (upper group), one falciger with thick handle, blade short, twice as long as wide, partially serrated, first 1/3 of the blade serrated (Fig. 6I); unit B (median group), three falcigers with thick handles, blades medium-sized, 2–3 times as long as wide, completely serrated (Fig. 6J); unit C (lower group), three falcigers with thick handles, blades short, as long as wide, smooth (Fig. 6K); unit D (lowest group), one falciger with slender handle, blades short, as long as wide, smooth (Fig. 6L). Units from the first anterior segments only long slender falcigers (Fig. 6M). Ventral cirri short, 1/2 as long as neuropodia.

Posterior region slightly tapered, pygidium bilobular with two subulate cirri, short, as long as pygidial segment, anus terminal (Fig. 6A).

**Variation.** Paratype and additional material with pharynx fully everted with 18 lanceolate distal papillae and two subdistal lateral papillae, eyes barely seen; prostomium folded, and eyes could be partially concealed by first segments. Males with large masses of sperm in middle segments.

**Type locality.** Off Holbox (22°9′59.28″N, 87°12′59.10″W), Quintana Roo, Mexico.

**Distribution.** Tropical Northwestern Atlantic: Western Caribbean, from off Holbox to Nichupté Lagoon Quintana Roo, Mexico. The revision of the material examined by Taylor (1971), San Martín, Aguirre & Baratech (1986) and Pettibone (1992) might extend the distribution of *T. anabelae* sp. nov. to the Floridian ecoregion and to the Southern Gulf of Mexico.

**Etymology.** The name of this species is a humble recognition of the great labor done by Anabel León as a technician assistant in the Laboratorio de Biodiversidad y Cambio Climático (BIOMARCCA), ECOSUR-Campeche for many years. The epithet is a noun in the genitive case (ICZN, 1999, Art. 31.1.2).

**Remarks.** Before this study, the genus *Taylorpholoe Pettibone, 1992* was considered monotypic, with *T. hirsuta* (Rullier & Amoureux, 1979) as its only species. *Taylorpholoe hirsuta* appeared to have a wide distribution in the West Atlantic Ocean, from South Carolina, USA to Brazil (Pettibone, 1992); however, with *T. anabelae* sp. nov. taking place in the same region, the previous records of *T. hirsuta* should be carefully revised.

As both species belong to the genus *Taylorpholoe* the similarities between *T. hirsuta* and *T. anabelae* sp. nov. are noticeable; however, *T. anabelae* sp. nov. differs by having a notopodial stylode with a large wide basis and, elytra with consistently abundant fimbria, whereas *T. hirsuta* has short slender notopodial stylodes and elytra with sparse long fimbria. The main difference between both species is noted in the dorsal papillae. *Taylorpholoe anabelae* sp. nov. has larger and bulbous papillae, completely covering the dorsum, while *T. hirsuta* present small and flat papillae, only covering the middorsal line (Padovanni, 2014: 59, fig. 7).

Pettibone (1992) recombined *Pholoe minuta hirsuta* and elevated its status, using it as the type species of *Taylorpholoe*. She used material from the Tropical Northwestern Atlantic and beyond, including the type material from central Brazil. The redescription also included the material previously examined by Taylor (1971) from Florida, which fits the original description, and the first elytra from the holotype. However, the elytra did not match Taylor’s illustrations (1971: 115, figs. E–I), or drawings by San Martín, Aguirre & Baratech (1986: 13, fig. 5A), nor the illustrated for *T. anabelae* sp. nov. (Figs. 3V, 3W, 6E–6G and 7D). Type or topotype material of *T. hirsuta* should be used to properly complete the description of the species which will enable comparisons with congeneric species. Currently, the description of *T. hirsuta* made by Padovanni (2014) is the most comprehensive and well-illustrated description.

In addition, in the proposition of the genus *Taylorpholoe*, Pettibone (1992) recorded juveniles and adults of *T. hirsuta*; she recognized the smallest specimens with 11 segments as juveniles, and those with 14–19 segments and signs of oocytes or juveniles beneath elytra as adults. Padovanni (2014) revised 124 specimens with a range of 14–16 segments, and despite she did not specify if the specimens possessed oocytes or juveniles, it is possible that her specimens were adults. Here, the specimens examined had 13–15 segments, and all presented either sperm (ECOSUR 297), oocytes, or juveniles (ECOSUR 296; ECOSUR P3233).

**Subfamily Pisioninae Ehlers, 1901**

***Pisione* Grube, 1857**

***Pisione* Grube, 1857**: 174

**Type species.** *Pisione oerstedii* Grube, 1857 by monotype.

**Diagnosis.** Pisionins with prostomium reduced, surrounded by buccal segment (segment 1). Without antennae. Emergent buccal acicula might be present. Jaws present. Parapodia and chaetae present from segment 2. Neurochaetae of several kinds, simple chaetae, compound chaetae falcigers and spinigers. Copulatory apparatus temporary (after Yamanishi, 1998; Gonzalez, Worsaae & Eibye-Jacobsen, 2022).

***Pisione wolfi* San Martín, López & Núñez, 1999**

(Figures 8 and 9)

**Figure 8:**
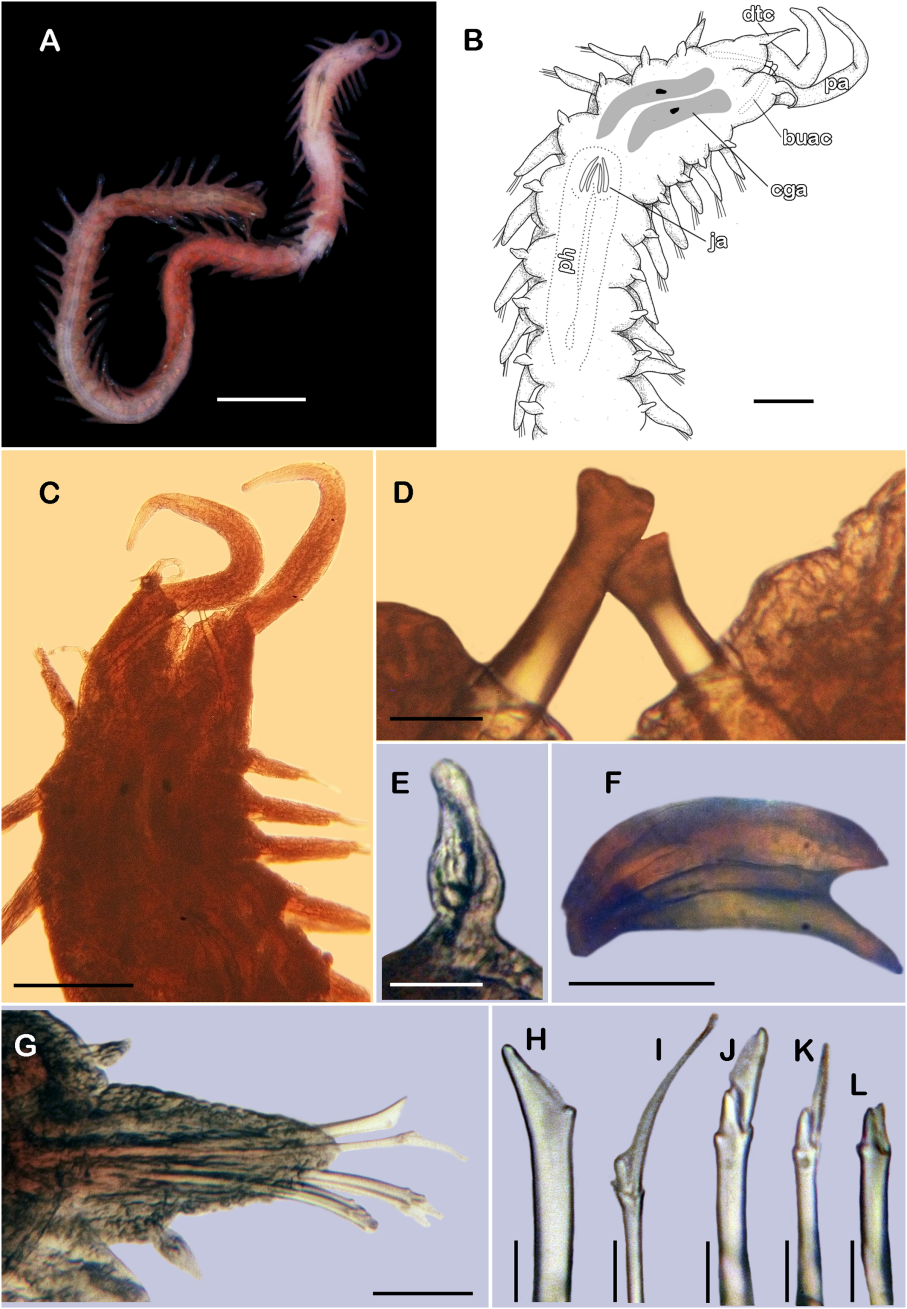
*Pisione wolfi* San Martín, López & Núñez, 1999 (non-type material ECOSUR P3235). (A) Complete specimen, dorsal view. (B) Anterior end, dorsal view. (C) Prostomium, dorsal view. (D) Close-up of buccal aciculae. (E) Left ventral cirrus from buccal segment. (F) Jaws. (G) Parapodium from segment 17. (H) Simple neurochaeta. (I) Spiniger. (J) Superior falciger. (K) Low falciger. (L) Lowest falciger, blade lost. Abbreviations: buac, buccal acicula; cga, cerebral ganglion; dtc, dorsal tentacular cirrus; ja, jaws; ph, pharynx. Scale bars: A: 500 µm, B, C: 100 µm, F and G: 50 µm, D, E, H–L: 10 µm.

**Figure 9:**
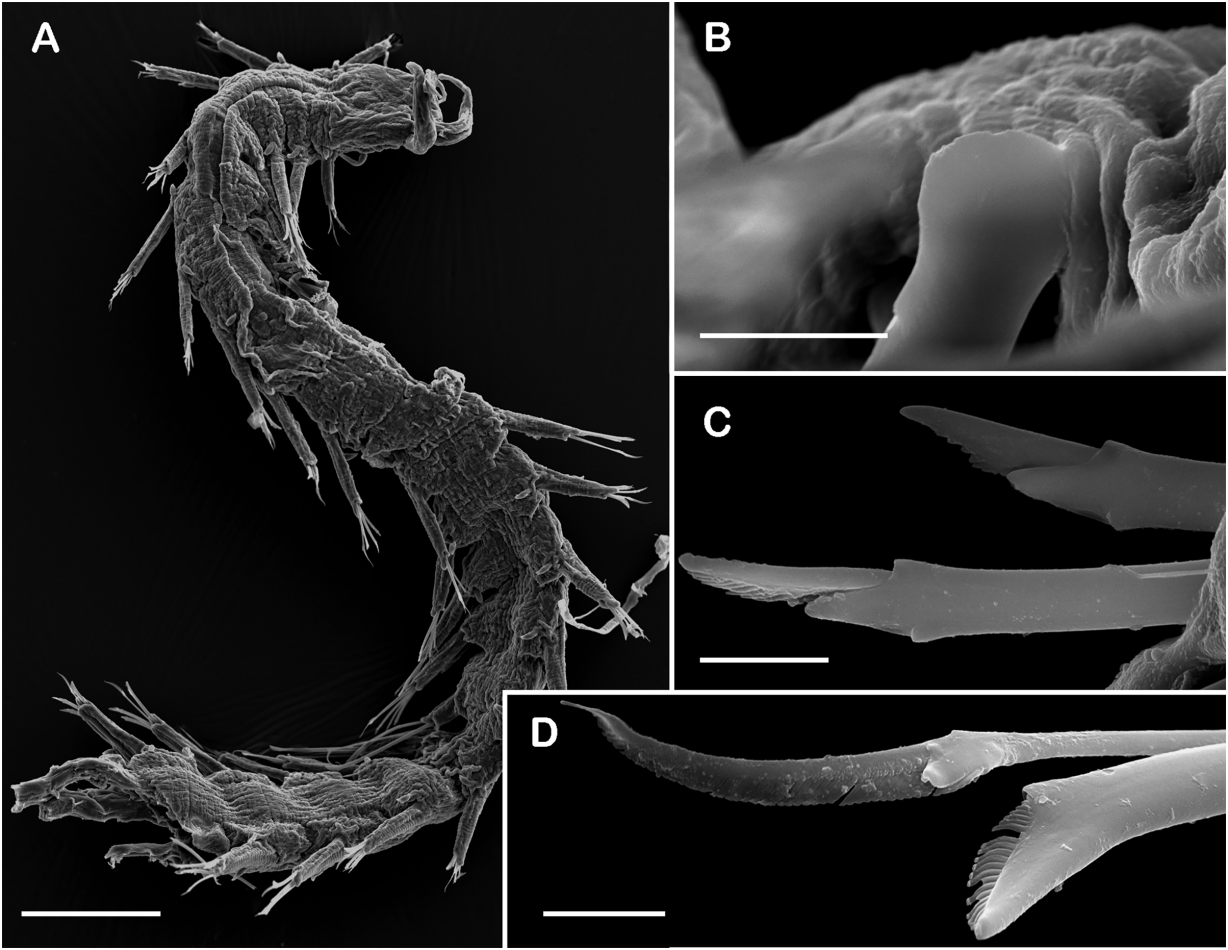
SEM micrographs of *Pisione wolfi* San Martín, López & Núñez, 1999 (non-type material ECOSUR P3234). (A) Anterior fragment, dorsal view. (B) Detail of buccal acicula. (C) Low and lowest falcigers from middle segment. (D) Spiniger and simple chaetae from middle segment. Scale bars: A: 200 µm, B–D: 10 µm. Photo credit: Sergio I. Salazar-Vallejo.

*Pisione* sp. A.—Wolf, 1984: 27-7, fig. 27-4.

*Pisione wolfi* San Martín, López & Núñez, 1999: 7, figs. 3–4.—Díaz Díaz, Bone & López-Ordaz, 2015: 125, figs. 2i–2n.

**Type locality.** Isla de la Juventud, Cuba.

**Material examined.**

**Mexico, Quintana Roo.** One specimen (ECOSUR P3234): off Isla Mujeres (22°4′0.22″N, 86°55′0.11″W); O/V UAT 1 CIDIPORT; 29 m; coarse sand; August 28, 2016; collected by Anabel León (coated in gold, anterior fragment, 13 segments, 0.2 cm long, 0.08 cm wide]; one specimen (ECOSUR P3235): off Isla Mujeres (21°18′0.79″N, 86°34′0.37″W); O/V UAT 1 CIDIPORT; 29 m; coarse sand; August 28, 2016; collected by Anabel León [fragment without posterior region, 51 segments, 0.8 cm long, 0.5 cm to segment 30, 0.02 cm wide).

**Description of non-type material (ECOSUR P3235).** Non-mature specimen, incomplete (Figs. 8A and 9A). Body slender, long, whitish. Integument shiny, dorsal segmental furrow well-marked, ventrally barely developed (after staining, segmental furrow visible along body) (Fig. 8B).

Prostomium reduced, rhomboid, surrounded by buccal segment (Fig. 8C). Buccal segment quadrangular, as long as wide; dorsal cirri slender, eight times as long as wide; ventral cirri flask-shaped, short, 1/4 as long as dorsal cirri (Fig. 8E); palps thick, smooth, 10–11 times as long as wide. Eyes small, two pairs fused, inserted in segment three. Cerebral ganglia extended from buccal segment to posterior margin of segment five. Buccal acicula translucent, thick, oblique, extended along buccal segment, protruding, expanded distally, distal plate smooth (Figs. 8D and 9B).

Pharynx with two pairs of jaws, visible between segments five and six (Fig. 8F). Dorsal cirri small throughout body, cirrophores globular to oval, cirrostyles globular short, half as long as cirrophore, tips ciliate. Ventral cirri from first segment (buccal cirri) slender and long, five times as long as dorsal one, subsequent ventral cirri similar to dorsal ones, slightly smaller.

Segment 2 shorter than following ones, 3/4 as long as the succeeding one (Figs. 8B and 8C). Parapodial lobes truncated, prechaetal lobe entire rounded. Segments 3–6 slightly larger than following ones.

Right parapodium from segment 17 (middle segment), sesquiramous, notopodia reduced. Notacicula thick, short, 1/2 as long as neuracicula, neuracicula thicker (Fig. 8G). Neurochaetae include four kinds, in dorso-ventral sequence: one superior simple chaeta, distally obliquely swollen with about 21 thick spines on margin, with a subdistal globular tooth (Figs. 8H and 9D). Subsequent chaetae, only compound heterogomphs. One spiniger with handle slender, smooth, with distal scale, blade distally bent long, 10–11 times as long as wide, with about 51 thick short spines (Figs. 8I and 9D). Lower, three falcigers with handles thick with one subdistal tooth; blades straight, short, three times as long as wide, with about 7–10 thick short spines (Figs. 8J and 9C). Lowest, two falcigers with handles slender, with one subdistal tooth; blades straight, short, 3–4 times as long as wide, with inconspicuous spines, barely noticeable (Figs. 8K, 8L and 9C).

Copulatory parapodia and organs not seen. Posterior region lost.

**Distribution.** Tropical Northwestern Atlantic: Western Caribbean, Greater Antilles, and Southern Caribbean, from Quintana Roo, Mexico to National Park Archipelago Los Roques, Venezuela.

**Remarks.** These specimens match the original description by San Martín, López & Núñez (1999); however, some subtle differences were detected. The specimens here examined show the cerebral ganglion slightly displaced posteriorly, covering from the mid-section of segment five to the posterior margin of the buccal segment, without touching the buccal acicula and the jaws were located between the segments five and six. On the other hand, San Martín, López & Núñez (1999: 32, fig. 1A) described that in their specimens the extension of the cerebral ganglion comprehended from the posterior section of the segment four to the peristomium, reaching the basis of the buccal acicula, and the jaws were described and illustrated at segment five. The number of chaetae per segment (except segment one) also varied. Here, the examined specimens have 6–7 chaetae per segment (one superior simple chaetae, one spiniger, three lower falcigers and two lowest falcigers), while *P. wolfi* was described with five chaetae per segment (one superior simple chaetae, one spiniger, and three lower falcigers) (San Martín, López & Núñez, 1999), the same number of chaetae in the *P. wolfi* recorded by Díaz Díaz, Bone & López-Ordaz (2015) from National Park Archipelago Los Roques, Venezuela.

*Pisione wolfi* was described using mature male specimens, whereas here the examined specimens are incomplete and possibly immature, as deduced by the lack of copulatory organs or gametes.

## Discussion

This study increases the knowledge of the sigalionid diversity of the Tropical Northwestern Atlantic with the confirmation of the record of *Pisione wolfi* in the region, and the description of three new species. The new species fit within the genera *Sthenelanella* (*S. pechi* sp. nov. and *S. sarae* sp. nov.) and *Taylorpholoe* (*T. anabelae* sp. nov.).

Since the erection of *Sthenelanella* over 100 years ago, only six species have been described; back then, using features in the body, parapodia, and elytra. Subsequent redescriptions, such as the ones made by Pettibone (1969), have broaden the descriptions of the species by evaluating in detail the prostomial features, first anterior segments, elytral features and the diverse kind of chaetae. This initiative for more detailed descriptions was also followed by Thomassin (1972), Lana (1991), Imajima (2003), Wehe (2007) and Aungtonya & Eibye-Jacobsen (2013). Here, the descriptions of the new *Sthenelanella* species have attempted to cover the classical features as well as the attributes included in recent studies, to make the descriptions comparable among the known species of the genus.

Some of the most contrasting attributes among *Sthenelanella* species are found in the elytra, such as their shape and pigmentation pattern. The specimens of the two new species of *Sthenelanella* agree with two of the three kinds of pigmentation patterns recognized by Imajima (2003): transversally banded (*S. pechi* sp. nov. and *S. sarae* sp. nov.) and pigmentation mottled (*S. sarae* sp. nov.). Further, it should be noted that the site where the pigmentation occurs and its coverage are also diagnostic and discriminant among species.

Regarding notochaetae, recently Tilic et al. (2021) explored the so-called thread-like fibers, hairy or feltage notochaetae of *Sthenelanella* by studying the process of chaetogenesis and ultrastructure of the chaetae, concluding that these are fibrous feltage chaetae, that emerge from modified chaetal sacs. Several authors have stated that the bundles of feltage chaetae appear in the middle segments, usually between segments 14–16 (Hartman, 1939; Fauvel, 1958; Lana, 1991; Imajima, 2003; Tilic et al., 2021). Here, the same segments were dissected and described from specimens of *S. pechi* sp. nov. and *S. sarae* sp. nov.; unfortunately, the chaetal sacs went missing during mounting on the slides, but were observed while dissecting the segments. All the *Sthenelanella* specimens examined herein had only a few short feltage chaetae, and none was collected with a fibrose tube. It is important to note that the specimens were collected from sediment by nucleator sampling from the sea bottom, which could result in chaetae being lost during the sampling; or perhaps, the interstitial habitat where the specimens were found led to the loss of long chaetae that might prevent free displacement among sand grains.

Regarding the neurochaetae, the chaetal unit classification proposed by Cruz-Gómez (2022) for pelogenins has been also proven useful for the genera *Sthenelanella* and *Taylorpholoe*, especially for descriptions and comparisons.

Unlike parapodia of pelogenins, sthenelanellins and pholoins have lesser complex parapodia, which is reflected in the distribution of the neurochaetae in the neuropodia. However, some similarities and differences regarding the neurochaetae distribution pattern were noted among the subfamilies, based on the species here examined. The similarities among the species evaluated are in the site of insertion of the units. In the three subfamilies, the neurochaetae of unit A (upper group) are always inserted above the neuracicula; neurochaetae of unit B (median group) are inserted above, beneath, and below neuracicula; and neurochaetae of units C (lower group) and D (lowest group) are always inserted below the neuracicula in that order. On the contrary, the pattern of insertion of the neurochaetal units in the neuropodia differs among subfamilies; while pelogenins present complex patterns such as marked “C” shaped and “J” shaped patterns of insertion in the neuropodia, sthenelanellins and pholoins present inconspicuous “C” shaped and “J” shaped patterns.

The description and illustrations of the neurochaetae of *Sthenelanella* species have been heterogenous among studies. Few studies include the characterization of some neurochaetae from middle segments (*e.g*., Fauvel, 1958); some others have described the neurochaetae briefly by distinguishing the articulate blade from anterior segments and the blunt short blade from middle segments (*e.g*., Aungtonya & Eibye-Jacobsen, 2013). In such studies, the descriptions were complemented with detailed drawings of the neurochaetae. The proposed classification of neurochaetae by units provides another option to present the descriptions regarding neurochaetae. Using this terminology might help to generate less verbose descriptions, cover all the variety of chaetae and, make descriptions comparable among species (*sensu* Cruz-Gómez, 2022).

The genus *Taylorpholoe* was considered monotypic for the past 30 years, yet with the description of *T. anabelae* sp. nov. this changed. The recognition and description of *T. anabelae* sp. nov. was possible by considering uncommon features used for sigalionids, but explored in other annelid families (*i.e*., Sphaerodoridae Malmgren, 1867), such as the distribution, shape and coverture of dorsal papillae. The presence of dorsal papillae was mentioned in previous descriptions of *Taylorpholoe* specimens (Wolf, 1984; San Martín, Aguirre & Baratech, 1986; Pettibone, 1992); however, in those studies the authors did not evaluate the taxonomical relevance of these dorsal structures. Here, the dorsal papillae were considered taxonomically informative, and they proved their utility to separate the newly described species *T. anabelae* sp. nov. from the known *T. hirsuta*. Likewise, the elytra in this genus are taxonomically useful as in any scaled sigalionid; however, while studying the shape of the papillae they should be assessed carefully, since they might suffer a distortion caused by the accumulation of dirt on their cilia (V. Miranda, 2021, personal communication). The marginal papillae found in *T. anabelae* sp. nov. might be perceived as flask-shaped, similar to the ones illustrated by Taylor (1971: 115, figs. H–I); however, once the elytra are properly clean, the papillae get its actual shape, filiform covered with cilia (Figs. 7V and 7W). As for the examined specimens of *Sthenelanella*, neurochaetae of specimens of *T. anabelae* sp. nov. were described using the chaetal unit classification obtaining similar results. In *T. anabelae* sp. nov. the neurochaetae distribution in middle segments consists of two main patterns: the units A–C forming a continuous “C” shaped pattern surrounding the neuracicular lobe in dorsal to ventral direction, and unit D, inserted in a wide “U” shaped pattern on the ventral side of the parapodia, starting from the anterior side of the parapodia to the posterior side. In contrast, in some genera of Pelogeniinae (*i.e*., *Daypsammolyce* Pettibone, 1997, *Hartmanipsammolyce* Pettibone, 1997, *Neopsammolyce* Pettibone, 1997, *Pelogenia* Schmarda, 1861, *Psammolyce* Kinberg, 1856) the neurochaetal units are presented in different patterns of interrupted fascicles; for instance, in *Pelogenia* the unit A forms a “J” shaped pattern, while the units B and C form a continuous “C” shaped pattern surrounding the neuracicular lobe, if secondary chaetal units are present they follow the previous “C” shaped pattern, and finally unit D comprehend a wide “U” shaped pattern on the ventral side of the parapodia (Cruz-Gómez, 2022: 4, figs. 1H–1J).

Finally, this study involved the revision of specimens collected from underexplored sites and depths in the Tropical Northwestern Atlantic. Unfortunately, because they were fixed in formalin solution, it was not possible to complement the species descriptions with DNA sequences. However, the lack of molecular information did not impede the determination of the species identities, both in previously described and new species.

Little effort had been made to study these subfamilies of sigalionids in the region; nevertheless, as the studies of these groups increase, the hidden diversity of these small worms is being revealed.

## Conclusions

The subfamilies, Pholoinae, Sthenelanellinae and Pisioninae, are three more-or-less common subfamilies of sigalionids. In the Tropical Northwestern Atlantic, study on these subfamilies has been scarce with only eight species recorded, including questionable records for the region. In this study, three new species were described from this tropical region; the description included detailed images, such as SEM imaging, photographs, and drawings, as well as the use of the unit terminology for neurochaetae recently proposed. The identification keys included herein will help to assist in taxonomical, or ecological studies. In the region as in other oceanic basins, more efforts should take place to increase the knowledge of these sigalionid subfamilies. Along with this work, the result of future surveys focused on these groups of small worms will become an important information input for the study of the phylogeny of the family Sigalionidae.

## Additional Information and Declarations

### Data Availability

The following information was supplied regarding data availability: The raw data related to the specimens is available in the ‘material examined’ text. The specimens are available in the Scientific Collection of El Colegio de la Frontera Sur, Chetumal: ECOSUR P3232, ECOSUR P3234, ECOSUR P3235, ECOSUR 296, ECOSUR 297, ECOSUR 293, ECOSUR 294, ECOSUR 295.

### New Species Registration

The following information was supplied regarding the registration of a newly described species: Publication LSID: urn:lsid:zoobank.org:pub:D8DB22A2-E239-4CAD-A34C-B3B12B17E1B9 *Sthenelanella pechi* LSID: urn:lsid:zoobank.org:act:316A956C-51A4-4B18-A747-912454361D4B *Sthenelanella sarae* LSID: urn:lsid:zoobank.org:act:996882CD-2879-4107-B4D1-E4241E86C361 *Taylorpholoe anabelae* LSID: urn:lsid:zoobank.org:act:802B8684-3C7E-4D0E-8F25-17AF3E21D406
